# Comparison of mechanical properties and host tissue response to OviTex™ and Strattice™ surgical meshes

**DOI:** 10.1007/s10029-023-02769-0

**Published:** 2023-04-08

**Authors:** J. Lombardi, E. Stec, M. Edwards, T. Connell, M. Sandor

**Affiliations:** Allergan Aesthetics, an AbbVie Company, 4 Millennium Way, Branchburg, NJ 08876 USA

**Keywords:** Biologic mesh, Hybrid mesh, Hernia, Acellular dermal matrix, Rodents, Primates

## Abstract

**Purpose:**

This study compared the in vitro/benchtop and in vivo mechanical properties and host biologic response to ovine rumen-derived/polymer mesh hybrid OviTex™ with porcine-derived acellular dermal matrix Strattice™ Firm.

**Methods:**

OviTex 2S Resorbable (OviTex 2S-R) and Strattice morphology were examined in vitro using histology and scanning electron microscopy; mechanical properties were assessed via tensile test; in vivo host biologic response and explant mechanics were evaluated in a rodent subcutaneous model. Separately, OviTex 1S Permanent (OviTex 1S-P) and Strattice were evaluated in a primate abdominal wall repair model.

**Results:**

OviTex 2S-R demonstrated layer separation, whereas Strattice retained its structural integrity and demonstrated higher maximum load than OviTex 2S-R out-of-package (124.8 ± 11.1 N/cm vs 37.9 ± 5.5 N/cm, *p* < 0.001), 24 h (55.7 ± 7.4 N/cm vs 5.6 ± 3.8 N/cm, *p* < 0.001), 48 h (45.3 ± 14.8 N/cm vs 2.8 ± 2.6 N/cm, *p* = 0.003), and 72 h (29.2 ± 10.5 N/cm vs 3.2 ± 3.1 N/cm, *p* = 0.006) following collagenase digestion. In rodents, inflammatory cell infiltration was observed between OviTex 2S-R layers, while Strattice induced a minimal inflammatory response. Strattice retained higher maximum load at 3 (46.3 ± 27.4 N/cm vs 9.5 ± 3.2 N/cm, *p* = 0.041) and 6 weeks (28.6 ± 14.1 N/cm vs 7.0 ± 3.0 N/cm, *p* = 0.029). In primates, OviTex 1S-P exhibited loss of composite mesh integrity whereas Strattice integrated into host tissue with minimal inflammation and retained higher maximum load at 1 month than OviTex 1S-P (66.8 ± 43.4 N/cm vs 9.6 ± 4.4 N/cm; *p* = 0.151).

**Conclusions:**

Strattice retained greater mechanical strength as shown by lower susceptibility to collagenase degradation than OviTex 2S-R in vitro, as well as higher maximum load and improved host biologic response than OviTex 2S-R in rodents and OviTex 1S-P in primates.

**Supplementary Information:**

The online version contains supplementary material available at 10.1007/s10029-023-02769-0.

## Introduction

Surgical meshes facilitate tissue repair by providing support to weakened areas following surgical procedures, including abdominal wall repair [1]. Traditionally, surgical meshes were classified based on composition as either synthetic or biologic [2]. Recently, with the emergence of new biomaterials and processing technologies, surgical mesh categories can be divided into permanent synthetic, resorbable synthetic, biologic, and hybrid meshes, the latter of which contain both biologic and synthetic components [2]. Although synthetic meshes are constructed from strong and affordable materials, their use is commonly associated with inflammation, scar formation, migration, and potentially erosion and pain [2–4]. Furthermore, synthetic meshes are not recommended in contaminated or infected fields due to the risk of complications resulting from infection [3, 5]. Unlike synthetics, biologic meshes are composed of soft tissue constructs, typically derived from human, porcine, bovine, or other mammalian tissues and processed in an attempt to preserve the extracellular matrix (ECM) while removing antigenic epitopes that may lead to host rejection [1, 6–9]. Biologic meshes provide a collagen-based scaffold that support host tissue cellular repopulation and revascularization to reinforce weakened abdominal tissue [1, 6–9]. However, these can be relatively expensive, and mechanical support may depend on the particular biologic mesh used, how it was processed, and the resulting effect on tissue remodeling [2]. Hybrid meshes, which combine synthetic and biologic constituents, were developed to offer the benefits of both, including increased mechanical strength, decreased inflammation, and improved host tissue integration within one device [2, 10, 11].

Strattice™ Firm is a biologic porcine-derived acellular dermal matrix indicated for use in hernia repair and other soft tissue defects [12]. Unlike crosslinked biologic mesh products, Strattice is non-crosslinked, which minimizes the encapsulation event that is typically triggered by the inflammatory responses to an implanted foreign body [7, 9, 13, 14]. OviTex is an ovine rumen-derived/polymer mesh hybrid also indicated for the repair of hernias and/or abdominal wall defects [15, 16]. Several material configurations have been developed and are commercially available with either a permanent (polypropylene) or resorbable (polyglycolic acid) polymer stitching reinforcing the multiple biologic material layers.

With the increasing number of available surgical mesh materials, comparative studies are needed to evaluate the material and mechanical properties (eg, pore shape and size, load, stiffness), as well as the interaction with surrounding host tissue (eg, inflammatory response, fibrosis, degradation, integration), which may provide an indication of clinical performance [17–19]. However, both clinical and preclinical comparative performance studies that evaluate the mechanical properties and host tissue responses to hybrid meshes are limited [10].

The main objective of this study was to compare the mechanical properties and host biologic response to OviTex 1S-Permanent (OviTex 1S-P) and OviTex 2S-Resorbable (OviTex 2S-R) with Strattice, using both in vitro and in vivo test methods.

## Methods

### Biomaterials

The following commercially available products were used in this study: Strattice (Allergan Aesthetics, an AbbVie Company, Branchburg, NJ), OviTex 1S-P and 2S-R (TELA Bio, Malvern, PA). Samples were derived from a single lot for each mesh type tested. Materials were prepared according to respective manufacturers’ instructions for use prior to all testing procedures [12, 15, 16].

### Study design

In vitro/benchtop and in vivo characterization were performed on test samples. For in vitro characterization, comparisons were made between Strattice and OviTex 2S-R. Scanning electron microscopy (SEM) and histology were conducted to evaluate out-of-package (OOP) morphology/structure. Tensile testing (OOP and after enzyme digestion) was performed to evaluate in vitro mechanical strength retention.

For in vivo characterization, the rodent subcutaneous implant model and primate abdominal wall repair model were utilized. Comparisons were made between Strattice and OviTex 2S-R for rodent studies and between Strattice and OviTex 1S-P for primate studies. Histopathology and tensile testing were performed on post-implanted samples to investigate host biologic response and in vivo mechanical strength, respectively.

### In vitro/benchtop characterization

#### Scanning electron microscopy

Mesh samples (1 × 1 cm) were fixed overnight in 2% glutaraldehyde, serially dehydrated in graded ethanol solutions (50–100%) for 30 min each, then treated overnight in 100% ethanol. Samples were further chemically dehydrated overnight using hexamethyldisilazane (Cat# 16700, Electron Microscope Sciences, Hatfield, PA) and left to air dry. Dried samples were gold sputter-coated and visualized on a Jeol JCM 5000 NeoScope™ (Jeol, Tokyo, Japan) at ×40 and ×100 magnifications.

#### Histology

Mesh samples (1 cm × 1 cm) were fixed for 24 h in 10% neutral buffered formalin (NBF), sectioned to a thickness of 5 µm, placed on glass slides, and stained with hematoxylin and eosin (H&E) to observe collagen integrity and ECM microstructure. Stained slides were observed under brightfield microscopy using a Nikon Eclipse microscope and Nikon NIS Elements BR imaging software (Nikon Instruments Inc, Melville, NY). Images were captured at ×40 to ascertain matrix structure and ×100 to observe host response.

#### OOP tensile testing

Mesh samples were mechanically tested as follows. Samples (*N* = 5, 1 cm × 6 cm) were placed in pneumatic side action grips with serrated grip faces at a gauge length of 4 cm and tensile tested at a controlled strain rate of 1.65 mm/min until failure on an Instron 5860 testing system (Norwood, MA) and maximum load/width (N/cm) recorded. Sample orientation yielding the highest maximum load was determined prior to definitive testing and used for all tensile samples in this study. Two rows of polymer stitching within each 1-cm wide OviTex sample were included to ensure mesh sampling uniformity.

#### Collagenase tensile testing

Mesh samples (*N* = 5, ~ 1 cm × 6 cm) were cut along the strongest orientation and incubated with excess collagenase enzyme from *Clostridium histolyticum* (Prod # C0130, Sigma Aldrich, St. Louis, MO). Briefly, 5 U/mL collagenase was prepared in 20 mM HEPES/5 mM calcium chloride solution. Samples were placed into individual 15 mL conical tubes with 10 mL collagenase solution each and incubated for 24 h, 48 h, and 72 h at 37 ℃ with gentle agitation, with fresh collagenase solution being replaced every 24 h. Samples were then tensile tested as stated above. Maximum load data were collected.

### In vivo rodent characterization

#### Subcutaneous implant model

Rats were fasted for 24 h pre-surgery (see Supplemental Information [SI] for more details on the animals). Animals were anesthetized with 3−4% isoflurane and maintained under 2% isoflurane during surgery. Carprofen (5.0 mg/kg) was administered subcutaneously. Once anesthetized, the dorsum region was clipped, and the surgical site was aseptically prepared using germicidal soap, 70% alcohol, and povidone iodine. Two horizontal incisions were created on either side of the spine in the cranio-caudal orientation and two pockets (~ 1 cm × 7 cm) were created using blunt dissection. Each animal was subcutaneously implanted without fixation with a pair of Strattice and OviTex 2S-R test materials (1 cm × 7 cm), and surgical sites remained separated and independent of each other. Incisions were closed with a 4–0 nonabsorbable suture.

All rats received carprofen (0.05 mg/kg) subcutaneously for 1–2 days post-surgery. At 3 or 6 weeks post-implantation, all rats were anesthetized using isoflurane, their implants removed, and then the rats were exsanguinated. Samples and surrounding tissue were placed in ice cold RPMI media prior to testing.

#### Histopathology

A 1 cm × 1 cm mesh piece was cut from one end of each sample explanted from the rodent subcutaneous model and was prepared for histologic analysis as above. Histology slides were stained with H&E. A blinded reviewer evaluated H&E-stained slides based on the level of cell infiltration, revascularization, inflammation, resorption, encapsulation, and integration with surrounding host tissues.

#### Mechanical testing

Host connective tissue was removed from the remaining 1 cm × 6 cm explanted mesh sample via blunt dissection. Samples were subjected to tensile testing in the same manner as above and maximum load (N/cm) recorded.

### In vivo primate characterization

#### Animals

Two cohorts each of 12 adult male cynomologus monkeys (Covance, Shanhai, China) were used (see SI for more details on the animals). Animals were randomized to receive either Strattice or OviTex 1S-P to be implanted within the abdominal wall, as previously described [7, 8, 20, 21]. For each test material, four animals were explanted at each time point (1, 3, and 6 months).

#### Abdominal wall repair model

Animals were fasted for up to 16 h before the procedure. Animals were anesthetized by intramuscular (IM) injection of ketamine (5–10 mg/kg), followed by Dexdomitor^®^ (0.033–0.075 mg/kg IM; Zoetis, Parsippany-Troy Hills, NJ), with additional ketamine or Dexdomitor given as needed throughout the procedure. Following anesthesia and surgical site preparation, a longitudinal mid-abdominal incision was made to expose an area of the abdominal muscle (~ 3 cm × 6 cm), and a bilateral longitudinal full thickness defect was created by removal of fascia, rectus muscle, and peritoneum. Defects were repaired with the appropriate test material that was equal in size to the defect (~ 3 cm × 6 cm). The implant was anchored at each of the four corners with a single interrupted polypropylene suture and secured to the edges of the rectus abdominal muscle and fascia in a continuous pattern also with polypropylene sutures. Subcutaneous tissues were closed with a continuous polydioxane suture, while the skin was closed with interrupted nylon sutures. Animals received IM injections of either flunixin meglumine for analgesia or similar nonsteroidal anti-inflammatory drug (2–5 mg/kg IM) and IM or subcutaneous injections of buprenorphine (0.03 mg/kg). Flunixin meglumine and/or buprenorphine for pain management was allowed twice daily for 3 days or longer post-surgery. Following surgery, animals were monitored daily, and wounds were examined for signs of inflammation or infection. Euthanasia was performed by intravenous overdose of sodium pentobarbital at either 1, 3, or 6 months post-implantation. Following euthanasia, the repair site was exposed, and the implanted graft and surrounding tissue grossly evaluated. The mesh and surrounding host tissue were excised by making an incision 2–3 cm to the outside circumference of the graft site. Explanted tissues were maintained in ice cold RPMI solution during transport prior to further evaluation.

#### Histopathology

A 1 cm × 1 cm piece from each sample was taken and cut in half on the diagonal. Half of the sample was placed into 10% NBF for histologic processing and embedded into paraffin blocks; the other half was placed into 20% sucrose in preparation for cryoembedding in optimal cutting temperature media and subsequent immunohistochemical staining. Slides were stained either histologically with H&E or immunohistochemically using antibody probes to the inflammatory cell marker CD-68 to detect macrophages (Cat# MA5-13324, Thermo Fisher Scientific, Waltham, MA). A blinded histopathology reviewer evaluated slides under brightfield microscopy for evidence of cell infiltration, revascularization, and inflammation using a Nikon Eclipse microscope and Nikon NIS Elements BR imaging software (Nikon Instruments Inc, Melville, NY).

#### Mechanical testing

Samples spanning the width of the repaired surgical defect sites, inclusive of newly deposited host tissues, were collected and tensile tested as stated above. Maximum load/width was recorded.

### Statistical analyses

The Anderson Darling Normality test was used to assess normality of all data sets.

Comparisons between samples were analyzed for statistical significance using a two-sample t-test with a 95% confidence interval. Statistical analyses were performed using Minitab (State College, PA).

## Results

### In vitro/benchtop testing

#### OOP histology

**Fig. 1:**
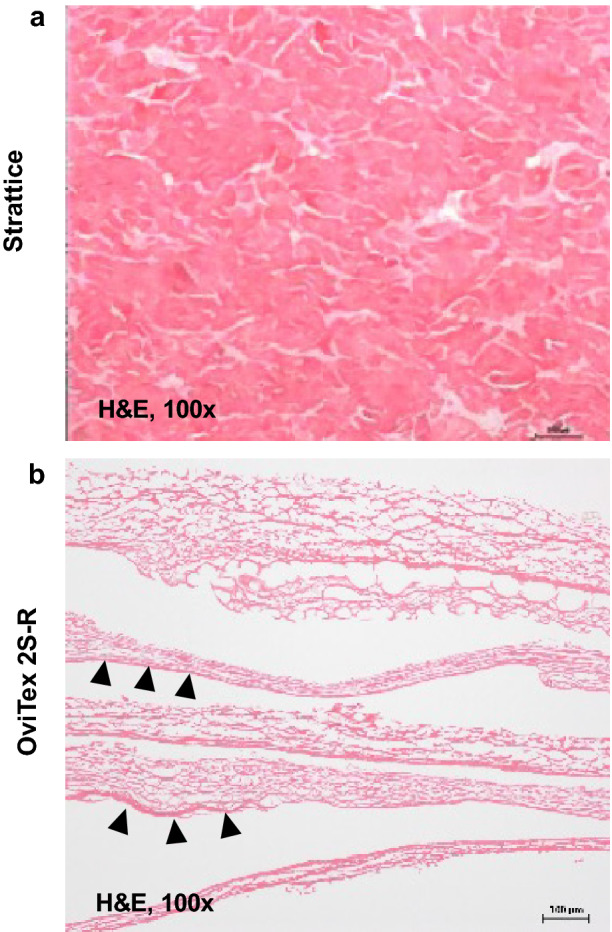
Representative hematoxylin and eosin (H&E)-stained images prior to implantation. **A** Strattice consisted of a dense layer of reticular collagen. **B** OviTex 2S-Resorbable (OviTex 2S-R) had less dense collagen than Strattice, as well as large void spaces. Each of the layers showed variable thickness. Arrowheads indicate the membranous side of the tissue layers, highlighting their anisotropy and possible differences in permeability. Images are shown at 100× magnification

Strattice porcine dermal collagen exhibited a singular, uniformly dense layer of reticular collagen throughout the entire thickness of the mesh. In contrast, OviTex 2S-R ovine rumen collagen was thinner and less dense and comprised spongy layers of variable thickness with large voids in-between layers (Fig. 1).

#### OOP scanning electron microscopy

**Fig. 2:**
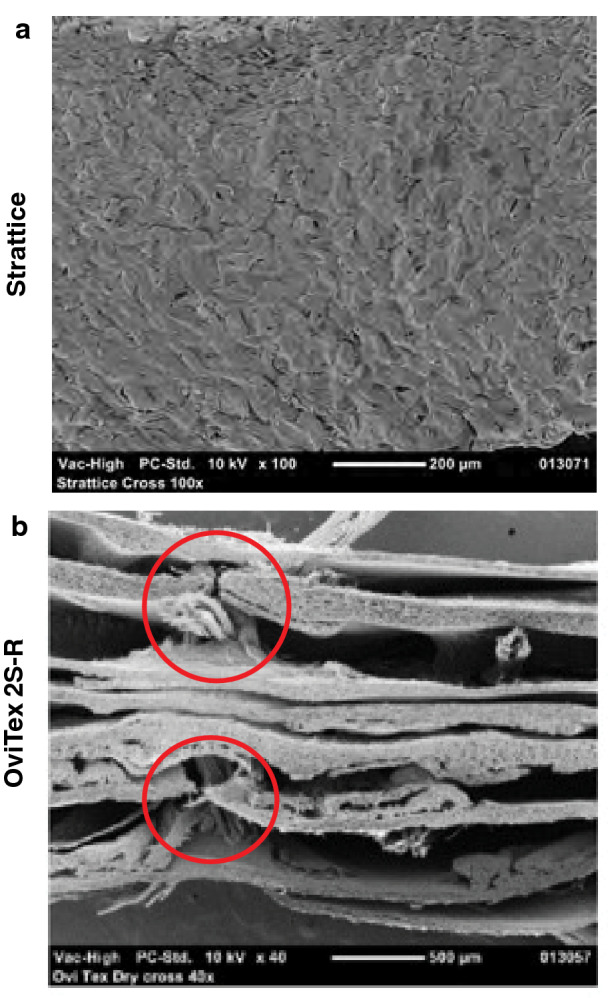
Representative scanning electron microscopy cross-sectional images prior to implantation. **A** Strattice showed an intact collagen matrix. **B** OviTex 2S-Resorbable (OviTex 2S-R) showed a porous collagen matrix. The multifilament polymer suture material of OviTex 2S-R (indicated by red circles) is evident between the separated biologic component layers. Micrographs are shown at 100×(Strattice) and 40×(OviTex 2S-R) magnification

Compared with Strattice, which demonstrated a thicker, more uniform reticulated collagen matrix structure (Fig. 2A), each of the 8 biologic layers of OviTex 2S-R demonstrated variable degrees of thickness (range, ~ 100–200 µm) consisting of an amorphous porous sponge-like pattern of thinner collagen bundles, with periodic penetration of multifilament polymer sutures (Fig. 2B). OviTex 2S-R also demonstrated noticeable layer separation, as well as separation between areas of compressed collagen within the biologic-derived layers, whereas void spaces and separation were absent in Strattice (Fig. 2B).

#### OOP and collagenase digestion tensile tests

**Fig. 3:**
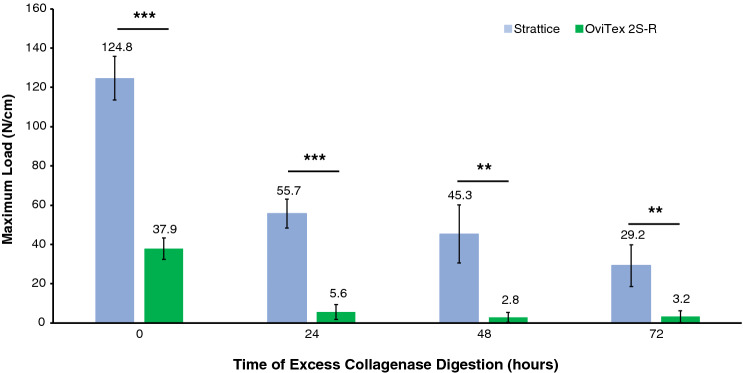
Results of in vitro tensile strength testing (maximum load, N/cm), both out-of-package (time 0) and following digestion via excess collagenase enzyme exposure. **p < 0.01, ***p ≤ 0.001

**Fig. 4:**
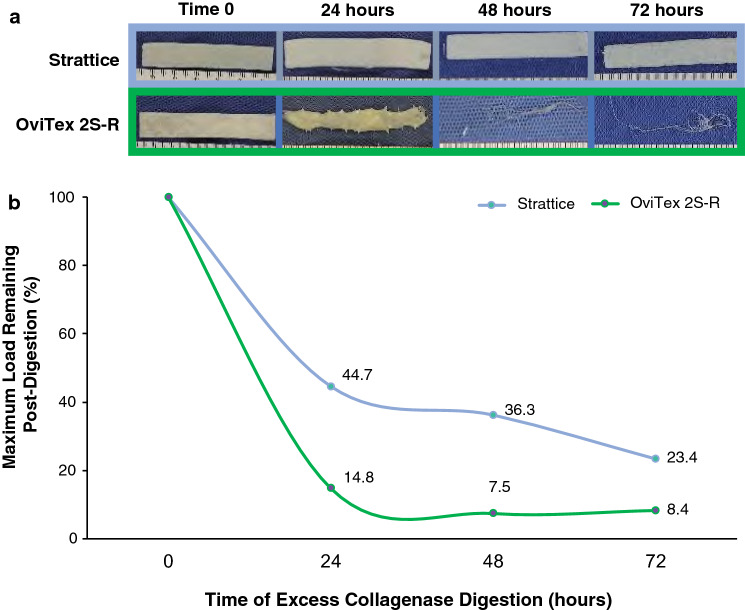
Strattice and OviTex 2S-Resorbable (OviTex 2S-R) over the time course of excess collagenase treatment. **A** Over the course of excess collagenase treatment, Strattice became slightly opaque, with hair follicles becoming apparent over time, but with the overall material remaining intact. OviTex 2S-R became degraded in the presence of collagenase, as shown by the loss in shape starting 24 h post-collagenase digestion and with the collagen component of OviTex 2S-R being completely digested by 48 h. **B** Strattice retained more of its initial strength measured as maximum load following a 24-, 48-, and 72-h collagenase digestion compared with OviTex 2S-R

Benchtop tensile testing showed that Strattice OOP was significantly thicker and had significantly higher maximum load than OviTex 2S-R OOP (1.5 ± 0.1 mm vs 1.1 ± 0.03 mm, 124.8 ± 11.1 N/cm vs 37.9 ± 5.5 N/cm,). Strattice also retained a higher maximum load at 24 h (55.7 ± 7.4 N/cm vs 5.6 ± 3.8 N/cm), 48 h (45.3 ± 14.8 N/cm vs 2.8 ± 2.6 N/cm), and 72 h (29.2 ± 10.5 N/cm vs 3.2 ± 3.1 N/cm) after in vitro excess collagenase digestion (Fig. 3). Forty-eight hours after collagenase digestion, the OviTex 2S-R biologic component had completely digested, leaving only the polymer component remaining (Fig. 4A). Strattice retained 44.7%, 36.3%, and 23.4% of its maximum load at 24-, 48-, and 72-h post excess collagenase digestion, respectively, compared with OviTex 2S-R that retained 14.8%, 7.5%, and 8.4% of its maximum load at the same time points (Fig. 4B).

### Rodent subcutaneous implant model

#### Histopathology

**Fig. 5:**
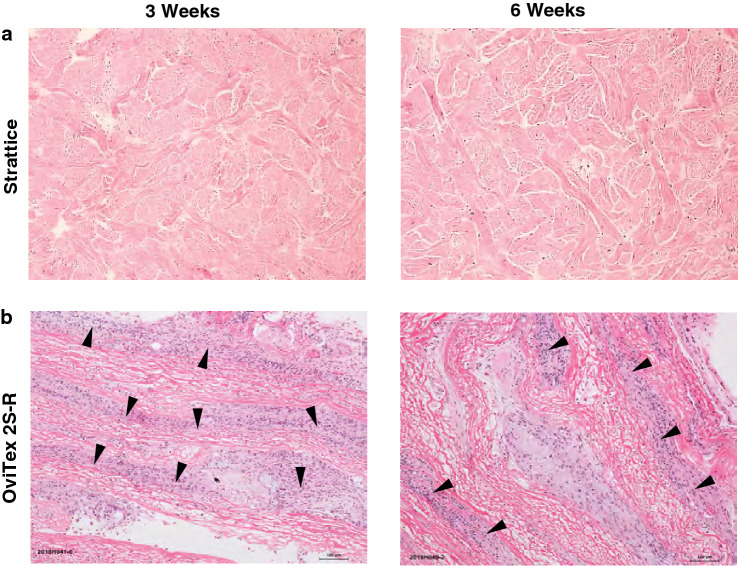
Representative hematoxylin and eosin images post-implantation in a rodent subcutaneous model. **A** Strattice showed minimal host inflammatory response. **B** OviTex 2S-Resorbable (OviTex 2S-R) demonstrated a considerable inflammatory response and separation of layers. Arrowheads indicate areas of inflammation. Images are shown at  × 100 magnification

For 3- and 6-week explanted rat samples, Strattice demonstrated minimal host inflammatory response, with notable infiltration of fibroblasts into the collagen tissue matrix (Fig. 5A). In contrast, OviTex 2S-R demonstrated considerable mixed inflammatory cell infiltration, predominantly between the tissue layers, resulting in separation of the biologic layers (Fig. 5B).

#### Mechanical testing

Strattice retained significantly higher maximum load at 3 weeks (46.3 ± 27.4 N/cm vs 9.5 ± 3.2 N/cm) and 6 weeks (28.6 ± 14.1 N/cm vs 7.0 ± 3.0 N/cm) post-implantation.

### Primate abdominal wall repair model

#### Gross pathology

**Fig. 6:**
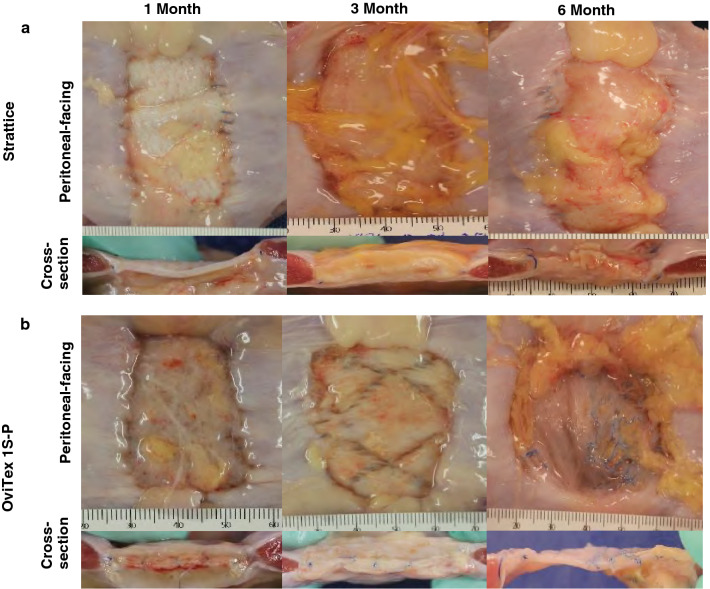
Representative macroscopic images of **A** Strattice and **B** OviTex 1S-P following 1, 3, and 6, months in a primate abdominal wall repair model. While Strattice appeared to integrate into the surrounding host tissue, OviTex 1S-P showed extensive biologic component resorption, resulting in synthetic component migration (ie, blue permanent polypropylene suture material) and loss of overall mesh integrity. For each test material, the top images show the peritoneal-facing surfaces, and the bottom images show the cross-sections

OviTex 1S-P demonstrated a gradual degradation of ovine-derived layers over time, resulting in loss of overall mesh integrity and subsequent contraction and migration of the synthetic component. In contrast, Strattice appeared to have maintained mesh integrity and to have integrated into the surrounding host tissue with minimal contraction (Fig. 6A). As early as 1-month post-implantation, OviTex 1S-P explants appeared thicker than OOP mesh due to a neoscar-like tissue layer covering both the dermal- and peritoneal-facing surfaces. There was also clearly visible separation of the ovine rumen biologic component layers (Fig. 6B). At 1-month post-implantation, Strattice explants exhibited minor folds and wrinkles and a slight increase in thickness due to a scar-like tissue layer covering only the dermal-facing surface of the implant (Fig. 6A)*,* which aligns with previously published findings in this model [8, 20–22].

#### Histopathology

**Fig. 7:**
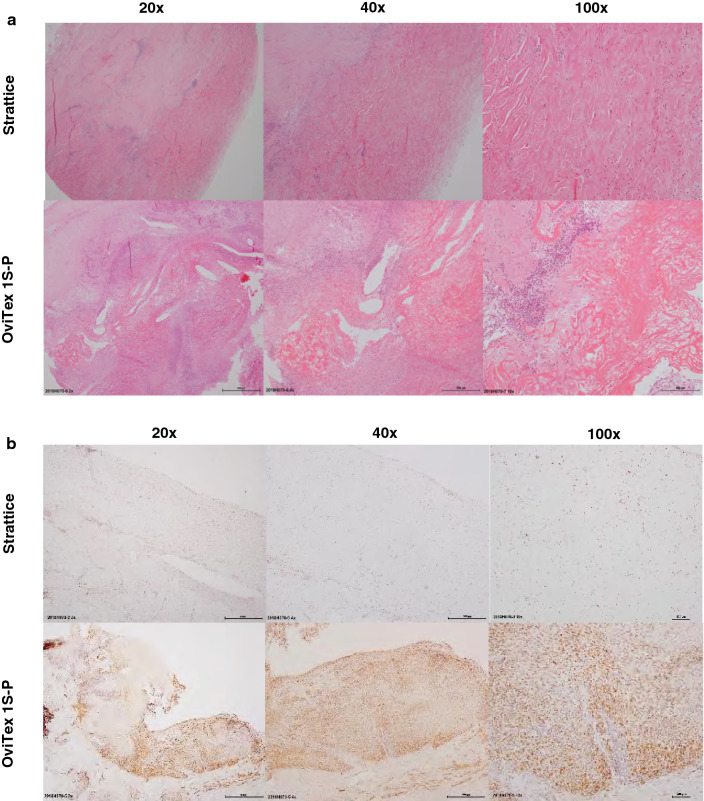
Representative histologic micrographs of surgical meshes explanted from a primate abdominal wall repair model (3 months post-implantation). **A** Hematoxylin and eosin images of Strattice showed diffuse cellular infiltration with evidence of collagen turnover, indicated by the lighter pink–stained areas (top images). OviTex 1S-Permanent (OviTex 1S-P) showed migration of the synthetic component away from the biologic components; the synthetic component is encapsulated by host tissue (bottom images). OviTex 1S-P also showed large areas of persistent inflammation. **B** CD-68-positive brown staining macrophages. Compared with Strattice, OviTex 1S-P showed robust host tissue inflammation. Images are shown at 20×, 40×, and 100× magnification

Strattice histology revealed diffuse fibroblast cellular infiltration, which occurred as early as 1-month post-implantation, and minimal to mild inflammation. OviTex 1S-P demonstrated a robust inflammatory response, further identified by inflammatory cell markers (CD-68^+^ macrophages) predominantly between mesh layers and resulting in layer separation (Fig. 7). OviTex 1S-P also demonstrated dissociation of the polymer suture away from the biologic layer component (Fig. 7).

#### Mechanical testing

Explanted Strattice exhibited a higher average maximum load at 1 month compared with OviTex 1S-P (66.8 ± 43.4 N/cm vs 9.6 ± 4.4 N/cm; *n* = 3 samples per group, *p* = 0.151). Direct comparisons between meshes could not be made at 3- and 6-months post-implantation because insufficient OviTex 1S-P samples were collected for testing (due to excessive in vivo contraction observed at these time points). However, Strattice implanted for 6 months in this model was shown to retain 77.5 ± 62.6 N/cm maximum load at the conclusion of the study.

## Discussion

The use of surgical meshes and their associated potential complications have recently come under close scrutiny by patients, healthcare agencies, and even government representative bodies, underscoring the need for adequate education and comparative studies [23]. Such studies are especially important for informing the selection of an appropriate mesh in the clinical setting where reduction of postoperative pain, stiffness, and other complications at the surgical site is paramount for hernia patients [1, 21, 24]. In concept, newly offered hybrid meshes should combine the desirable properties of synthetic and biologic meshes, with the synthetic component providing mechanical strength and the biologic component supporting host tissue integration, minimizing inflammation, and increasing resistance to infections [2, 10, 11]. However, the current study demonstrated that the biologic mesh Strattice retained greater strength over time than the hybrid meshes OviTex 1S-P and 2S-R, both in vitro and in vivo. Furthermore, Strattice demonstrated a more favorable biologic response than OviTex 1S-P and 2S-R, as indicated by greater fibroblast cellular infiltration and less inflammation. The overall architecture of OviTex 2S-R, created by the arrangement of several biologic layers and multifilamentous polymer suture used to secure the layers together yields interstitial void spaces, potentially increasing susceptibility to fluid accumulation and bacterial colonization. This observation aligns with a previously published study using a rabbit bacterial inoculation model, which reported high levels of bacterial colonization in OviTex 1S-P [10]. In the current study, OviTex 1S-P also showed evidence of dissociation of the synthetic sutures away from the biologic layer component due to collagen degradation, contraction of the synthetic sutures, and a lack of host tissue integration by 6 months. These results appear to contradict the original intent of the hybrid mesh concept, which is to maintain a permanent durable mesh component at the repair site. Strattice was less susceptible to in vitro degradation by collagenase than OviTex 2S-R, contributing to its greater retained strength. The rodent biomechanical study herein corroborated these results, confirming that Strattice retained substantially higher strength post-implantation compared with OviTex 2S-R. Compared with Strattice, OviTex 1S-P and 2S-R samples exhibited more host tissue inflammation, located predominantly between the biologic layers and resulting in their separation from one another in both the non-human primate and rodent models, respectively. Layer separation in OviTex samples could not be overcome by the inclusion of the synthetic suture material in the hybrid mesh product in an attempt to hold the multiple layers of the mesh in apposition.

Compared with in vitro studies, in vivo models capture the natural anabolic and catabolic processes of the host, as well as the complexities of scaffold degradation and host tissue integration [25, 26]. Studies using Old World primates are useful because they exhibit similar immune and foreign body responses as humans, owing to their high level of genomic homology [2, 27]. In the primate abdominal wall repair model, Strattice demonstrated a higher average maximum load at 1-month post-implantation compared with OviTex 1S-P, suggesting that there may be clinically relevant advantages in using Strattice over OviTex 1S-P. Mechanical properties, such as higher strength over time, as well as decreased susceptibility to degradation under increased enzymatic conditions (in the case of an infection), may indicate that a material is maintaining its integrity for a longer duration, providing sufficient support to the native tissue as remodeling occurs [28].

Less inflammation and better integration with the host tissue improves the degree of tissue regeneration, decreases postsurgical pain, and may prevent further surgical intervention [17, 19]. A separate comparative study in primates, evaluating different surgical meshes [2] including Strattice and OviTex 1S-P, reported higher overall host cell infiltration with OviTex 1S-P compared with Strattice at 4 weeks post-implantation. However, general host cell infiltration may not be the best predictor of material performance. The biologic response to any material is a complex series of events in which the types of cells, as well as their spatiotemporal existence in relation to the implanted materials, are critically important factors to consider. In the current study, OviTex 2S-R demonstrated greater general cell infiltration compared with Strattice at earlier time points; however, these cells were primarily inflammatory in nature, and the predominantly inter-layer location of the inflammatory response resulted in early separation of the OviTex 2S-R layers from one another. The timing, intensity, and location of the inflammatory response to OviTex 2S-R observed in the current study is not consistent with the kinetics of a healthy, more naturally occurring healing response as was seen with Strattice. The current study included more functionally relevant analyses, such as retention of mechanical strength over time, which the previous study did not investigate [2]. The excess concentration of collagenase enzyme used prior to tensile testing is similar to other previously published studies [25]. Although this concentration may not be clinically relevant [25], these results still suggest that Strattice material retains greater strength over time when subjected to enzymatic digestion compared with OviTex 2S-R [25].

The rodent subcutaneous implant model had several limitations that may have affected the level of observed inflammatory response. Studies have shown that mesh-mediated inflammation is partly linked to mechanical forces exerted by the host physiology [29, 30] and that remodeling of biological materials is influenced by host-dependent mechanical tension that is usually limited in small animal models [29–32]. With this limitation in mind and to generate supplemental data in a more functional, mechanically loaded model, the two meshes were also implanted in a well-established non-human primate abdominal wall repair model, which has been extensively used to evaluate host biologic responses to surgical meshes [8, 20–22]. Another limitation of the study was the use of animal models under healthy conditions. In the future, comparative studies involving functional or stress conditions (eg, infection/contamination) may be more clinically relevant [2, 33].

## Conclusion

Strattice elicited an overall preferable host biologic response compared with OviTex 2S-R in rodents and 1S-P in primates as demonstrated by less inflammation and greater retention of mesh integrity, which translated to greater retained mechanical strength in both models. This finding was corroborated by in vitro testing wherein Strattice retained greater strength over time than OviTex 2S-R hybrid mesh as shown by lower susceptibility to collagenase digestion.

## Supplementary Information

Below is the link to the electronic supplementary material.

Supplementary file1 (DOCX 15 KB)

## Data Availability

AbbVie is committed to responsible data sharing regarding the clinical trials we sponsor. This includes access to anonymized, individual, and trial-level data (analysis data sets), as well as other information (eg, protocols, clinical study reports, or analysis plans), as long as the trials are not part of an ongoing or planned regulatory submission. This includes requests for clinical trial data for unlicensed products and indications. These clinical trial data can be requested by any qualified researchers who engage in rigorous, independent, scientific research, and will be provided following review and approval of a research proposal, Statistical Analysis Plan (SAP), and execution of a Data Sharing Agreement (DSA). Data requests can be submitted at any time after approval in the US and Europe and after acceptance of this manuscript for publication. The data will be accessible for 12 months, with possible extensions considered. For more information on the process or to submit a request, visit the following link: https://www.abbvieclinicaltrials.com/hcp/data-sharing/.html.
