# Supplementary material for: Comparison of mechanical properties and host tissue response to OviTex™ and Strattice™ surgical meshes
Source: Hernia. 2023 Apr 8;27(4):987–97. doi: 10.1007/s10029-023-02769-0 (PMC10374700; doi:10.1007/s10029-023-02769-0)
Supplement: Supplementary file 1 — Supplementary file1 (DOCX 15 KB) [file 10029_2023_2769_MOESM1_ESM.docx]

# Supplementary Information

**Comparison of Mechanical Properties and Host Tissue Response to OviTex™ and Strattice™ Surgical Meshes**

Jared Lombardi, BS; Eric Stec, BS; Marianne Edwards, MS; Talia Connell, BS; Maryellen Sandor, PhD

## Rodents

Ten, 9- to 11-week old, immune-competent, male Lewis rats weighing 265−291 grams (Strain #004; Charles River, St-Constant, Quebec, Canada) were group-housed at the CHUM Research Center at University of Montreal, Montreal, Quebec, Canada and acclimatized for 5 days pre-study. Animals were fed ad libitum with standard rat chow and water. The experimental protocol was approved by the Institutional Animal Care and Use Committee of the University of Montreal.

## Primates

Twenty-four adult male cynomologus monkeys aged 4−7 years and weighing 4−7 kg (Covance, Shanghai, China) were quarantined for a minimum of 28 days and screened for general health before study entry. All animals were fed a primate diet twice per day. Testing was conducted at Allergan, plc., Irvine, California. All procedures were approved by the Allergan Animal Care and Use Committee.
